# First metatarsophalangeal fusion with dorsal plate: clinical outcomes

**DOI:** 10.1186/s13018-021-02453-y

**Published:** 2021-06-06

**Authors:** Giuseppe Restuccia, Fabio Cosseddu, Andrea del Chiaro, Matteo Ceccoli, Alessandro Lippi, Sheila Shytaj

**Affiliations:** Orthopaedic and Trauma Unit, University of Pisa, Via Paradisa 2, 56123 Pisa, Italy

**Keywords:** Arthrodesis, Hallux, Metatarsophalangeal joint fusion, Plating

## Abstract

**Background:**

First metatarsophalangeal joint (MTPJ) fusion is the most effective technique for the treatment of MTPJ primary arthritis, severe hallux valgus and failure of primary corrective surgery of these conditions. It can be achieved through different techniques. We evaluated the outcomes in a cohort of patients treated with dorsal plate arthrodesis.

**Materials and methods:**

We treated 30 feet for 28 patients; the mean follow-up was 35 months. For each foot, we collected radiological and clinical assessment, with the visual analogue scale (VAS) for pain and the Manchester and Oxford Foot questionnaire (MOFQ). The technique consisted in a cup and cone arthrodesis with the application of a low profile dorsal plate. Patients were allowed for immediate weight bearing.

**Results:**

Consolidation was achieved in all cases; in 29 cases, radiographic union was recorded within 6 months from surgery, in one case after 9 months. Comparison between the preoperative and postoperative of VAS and MOXFQ values showed a statistically significant difference (*p* < 0.05). Only one case developed wound dehiscence as complication.

**Conclusions:**

Even if there is still a debate regarding the best system for MTPJ fusion, we believe cup and cone fusion with dorsal plating is an effective method. Moreover, the stability of the osteosynthesis obtained allows for immediate post-operative weight bearing, making patients able to return soon to their normal life.

**Trial registration:**

We present a retrospective study; all patients enrolled were retrospectively registered.

## Background

The first metatarsophalangeal joint (MTPJ) fusion is a surgical procedure firstly described by Clutton in the last decade of 19th century [1]; despite of the several modifications introduced in the following years, it is still considered an effective technique for the treatment of several disorders of the first ray. The indications for fusion include MTPJ primary arthritis, severe hallux valgus or hallux varus and after failure of primary corrective surgery of these conditions [2, 3]. First MTPJ fusion aims to obtain a stable toe in order to reduce pain and discomfort. This can be obtained through different techniques ranging from the use of Kirchner wires to the application of staples, screws and plates [4]. Even when compared to alternative techniques—such as total joint replacement—first MTPJ arthrodesis seems to give far better clinical outcomes and lower rate of complications and reinterventions [5]. We report the clinical and radiological outcomes of a cohort of patients treated with dorsal plate arthrodesis without lag compression, allowed for immediate postoperative weight bearing.

## Methods

From January 2014 to January 2020, we treated 28 patients. The inclusion criteria were the following: primary MTPJ arthritis, hallux valgus deformity with advanced arthritis and hallux valgus relapse. Patients with previous MTPJ arthrodesis failure and those with history of first metatarsal or proximal phalanx fracture were excluded. Two patients had bilateral MTPJ fusion so the final number was 30 feet. The mean age of the sample was 69.5. The female male ratio was 24:2. The mean duration of the follow-up was 35 months, with no patients lost to follow-up. Eleven feet had primary MTPJ arthritis, 5 arthritic hallux valgus, 1 hallux varus and 13 hallux valgus relapse. All patients underwent preoperative and postoperative radiological and clinical evaluation. Standard anteroposterior (AP) and lateral weight bearing x-ray were performed before surgery, at 30 days and after 3 months. For the clinical assessment, we used the visual analogue scale (VAS) for pain and the Manchester and Oxford Foot questionnaire (MOFQ). Follow-up was carried at 2 weeks for wound inspection, than at 5 weeks, 3 months, and 12 months to evaluate clinical and radiological outcomes and complications. Union was defined both clinically, with pain recovery, and radiologically as the presence of bony bridging on 3 out of 4 cortices in at least one x-ray projection.

### Surgical technique

**Fig. 1:**
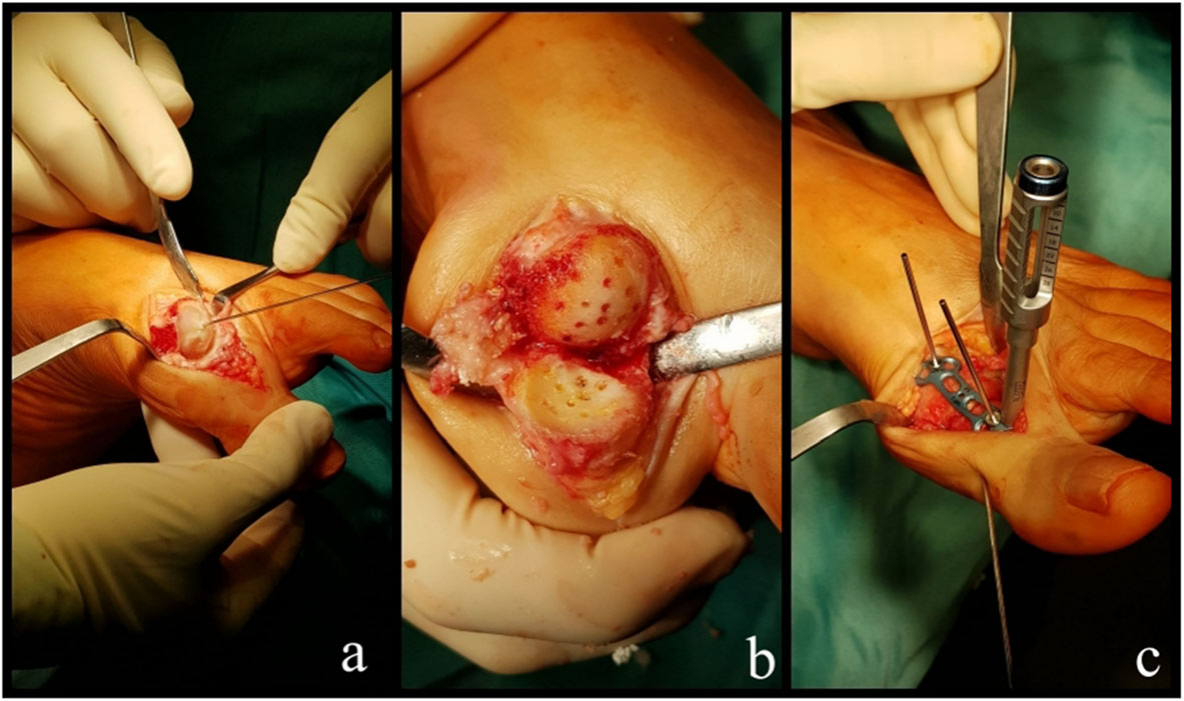
Surgical technique details. **a** Joint surfaces exposition and preparation. **b** Holes drilling on both surfaces. **c** Plate positioning

All procedures were carried out by the senior author with a standard operative technique and postoperative regimen. The patient was positioned supine in locoregional anaesthesia with leg tourniquet at 250 mmHg pressure. The approach to the MTPJ was dorsal extending from the midpoint of the proximal phalanx to the shaft of the first metatarsal. Protecting laterally the extensor hallucis longus tendon, the first MTPJ capsule was opened and the joint surfaces exposed and prepared with a system of cannulated cone and cup-shaped reamers. Two millimetres holes were drilled in the prepared surfaces and a proper debris was performed. The desired anatomical position was temporary maintained with a Kirschner wire and fluoroscopically checked with the foot on a plantar support. The arthrodesis was then fixed with a low profile dorsal plate with four locking screws 2.7 mm diameter (2 in the metatarsal and 2 in the phalanx); a compression screw was added on the plate if further stability was required, and it was in 5 cases (Fig. 1). After a final fluoroscopic check, the capsule was closed over the plate in order to protect the extensor tendon and the skin from it. The skin was closed with a 3-0 vicryl rapid suture and a bandage was applied. Immediate full weight bearing was allowed with a rigid sole shoe without high heel for 5 weeks.

### Statistical analysis

Statistical analysis was performed using the Statistical Package for Social Sciences, Version 13 (SPSS Inc., Chicago, Illinois). Continuous variables were showed as mean ± standard deviation, and discrete variables were expressed as frequency percentages. Student’s *t* test was used to analyse differences between pre- and post-operative clinical scores. For all the tests, we used a 5% level of confidence.

## Results

**Fig. 2:**
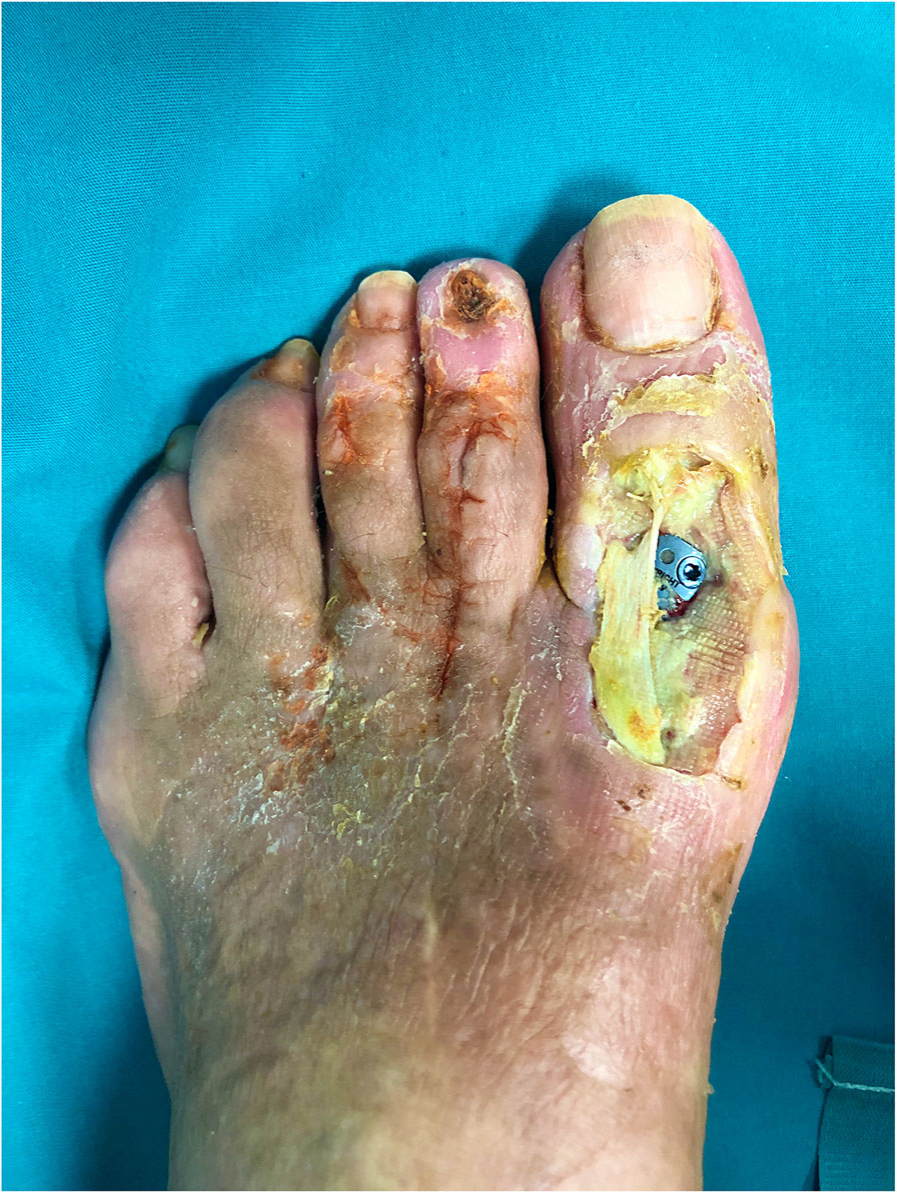
Wound dehiscence with plate exposure

**Fig. 3:**
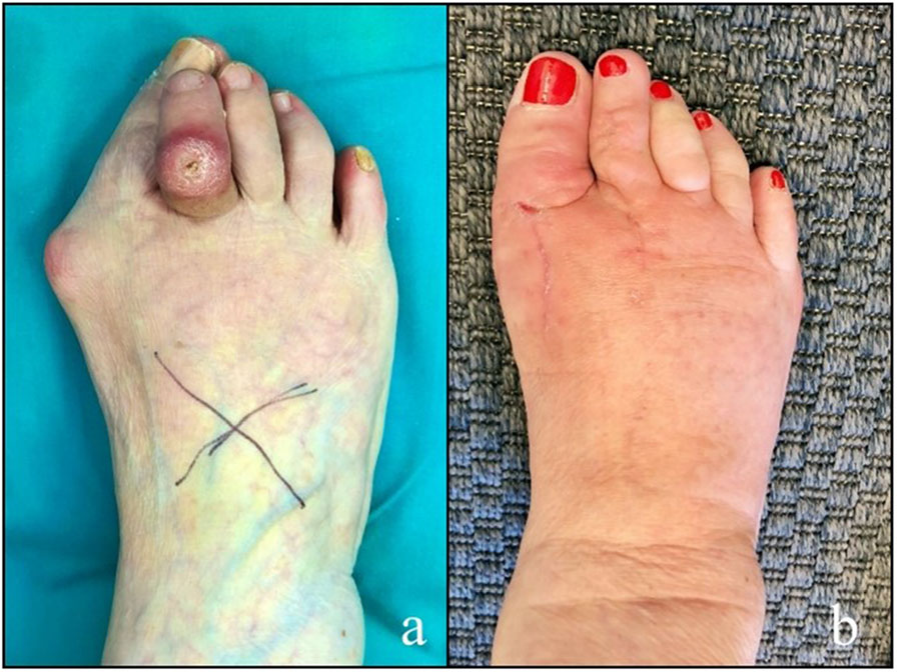
Clinical pre- (**a**) and post-surgical (**b**) aspect

**Fig. 4:**
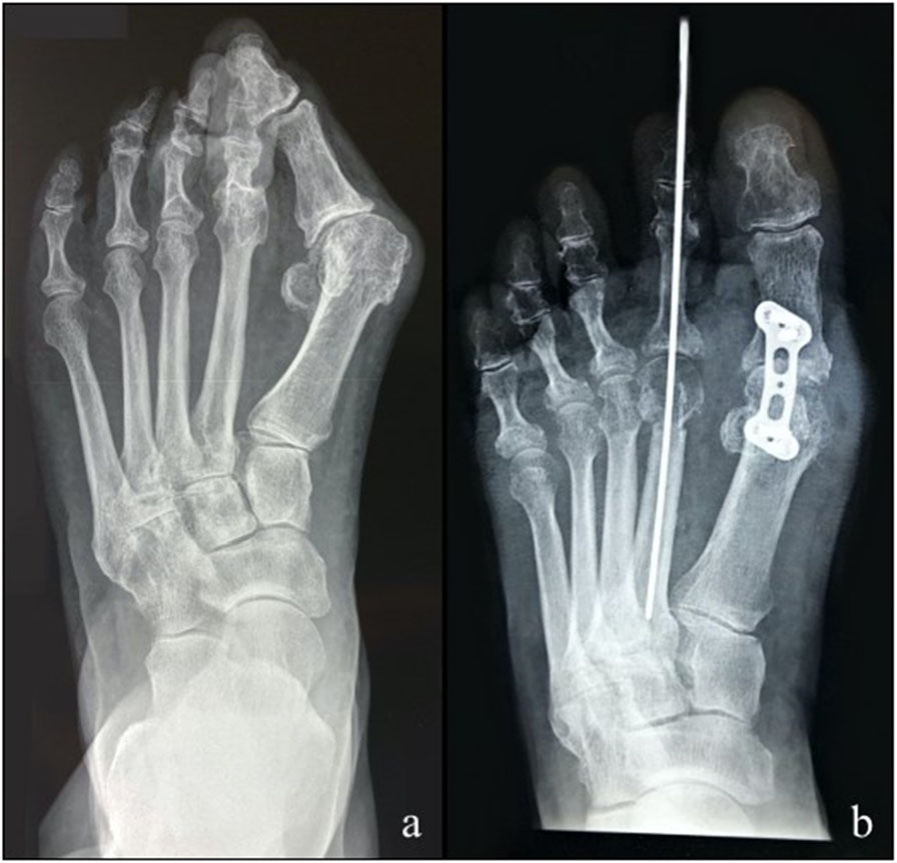
Radiological pre- (**a**) and post-surgical (**b**) aspect

Clinical union, considered as a VAS score < 3, was achieved at the second follow-up (after 4 weeks) in 29 cases. In all cases, except one, radiographic union was achieved within 6 months from surgery. One patient had a delayed union with radiographic healing at 8 months and clinical recovery at 3 months. The comparison between the preoperative and postoperative of VAS and MOXFQ values showed a statistically significant difference (*p* < 0.05). Mean preoperative VAS was 8.32 ± 1.14; instead, the post-operative was 1.48 ± 1.71. A significant reduction of MOXFQ score was noted too with a preoperative mean value of 47.44 ± 4.96 and a postoperative mean value of 2.92 ± 1.85. In one case, there was a wound dehiscence with deep infection that required plate removal after 2 months from the previous surgery (Fig. 2); the stability of the fusion was checked intra-operatively, and no revision of the arthrodesis was needed. No other soft tissue or bony complications were recorded. There were no cases of clinical discomfort related to the hardware (Fig. 3, Fig. 4).

## Discussion

Arthrodesis is rightfully considered the gold standard approach to end stage arthritis of the first MTPJ and to treat failed arthroplasty or hallux valgus surgery as a salvage procedure [2, 3, 6]. When performing a joint fusion is important to follow the Glissan principles of arthrodesis: careful joint debridement, alignment in optimal position, close fitting of the fusion surfaces and finally stability at the fusion site [7]. So far, several techniques have been described in order to achieve the optimal joint fusion, by varying the surgical approach, the resection technique or the fixation hardware. In our casuistry, we performed a dorsal open approach as most of the authors recommend having an optimal exposition of the joint without postoperative wound issues. Some authors advocate the plantar medial approach for the insertion of a lag screw; more recently, other authors have described percutaneous first MTPJ arthrodesis with some critical points regarding bone preparation and positioning [8, 9]. For the preparation of the articular surfaces, we used power-driven cannulated cup-cone reamers since they provide a wider contact surface allowing for higher adjustability of the final alignment; some authors still perform traditional flat cuts; however, they can bring some issues in positioning and finger shortening [10, 11]. The key to an optimal fusion is the position of the MTPJ since it eventually determines the effective function of the toe. The physiological alignment and load bearing capacity of the first ray should be taken into consideration. Some authors focus the attention on the relationship with second digit rather than to the primary orientation of the first toe [12]. However, there are commonly shared values in literature for position: 0° of rotation, 5° to 15° of valgus and 15° to 25° of dorsiflexion relative to the first metatarsal [13]. We respected these parameters, evaluating dorsiflexion intraoperatively by simulating weight bearing on a plantar support and with fluoroscopic check. When a satisfactory position is achieved, the fixation can be performed throughout different devices. Gibson et al. [14] described fixation with cerclage and Kirschner wire reporting an 83% fusion rate at 6 months; other authors reported the use of 2 crossed or parallel screws with better outcomes and non-union rates ranging from 0 to 9% [15–17]. The use of plates (locking, non-locking, alone or in association to other devices) is currently widespread [18, 19]. Politi et al. provided in 2003 a comparative biomechanical study between five techniques of fixation proving that the combination of a dorsal plate with a lag screw gives significantly more resistance to micromotion. He criticised the fixation with dorsal plate alone considering it as biomechanically disadvantageous since it is positioned opposite to the tensions side of the bone, which is the plantar one [20]. However, our outcomes go against this biomechanical explanation since all patients were treated with dorsal plate alone, with a single case of delayed union. Of course, the features of current plates are more sophisticated: the device we usually implant is a low-profile dorsal plate with four converging locking screws that provide an optimal fixation to the soft metatarsal and phalangeal bone stiffening the contact between plate and bone. However, if the construct needs more stability or the plate must be brought in closer apposition to the bone, a non-locking screw is added. More recently, Dening et al. reported a 90% rate of union for 2 screw fixation, plate and lag screw fixation and plate alone; indeed, in his study, the fixation with plate alone showed the lower non-union rate compared to the other techniques [21]. After an arthrodesis surgery in the lower limb, the recommendation was traditionally to keep patients non-bearing for a minimum of 6 weeks. Currently, there is no more consensus for this practice and the majority of authors are favourable to early postoperative weight bearing [4, 22]. We did not report any complication regarding the device or the bone allowing for immediate full weight bearing with a rigid sole shoe. The clinical evaluation was recorded with the MOFQ and the VAS score. The first is a 16-item questionnaire that measures the functional outcome while the second is a numeric pain score. For both evaluations, there was a drastic improvement comparing the pre and postoperative status. The results obtained confirm the literature data for arthrodesis, regardless of technique [4].

In spite of the optimal radiological and functional outcomes obtained, we believe that a wider group of patients is needed to definitely confirm these results. Also, a more detailed biomechanical evaluation of our technique of plating could be desirable, in order to explain the results achieved with the described technique. Moreover, even if our outcomes are very satisfactory, the study lacks a control group treated with another technique (i.e., plates and lag screw).

## Conclusions

Arthrodesis is a well-known procedure that usually gives satisfactory results in end-stage first MTPJ arthritis. Many techniques have been reported; however, in the last decade, there is actually a lack of studies describing the outcomes of first MTPJ arthrodesis with plate alone. We believe that the outcomes obtained with our technique could concur to show the validity of the dorsal plating with postoperative full weight bearing even if a wider cohort of patients would be desirable.

## Data Availability

The datasets used and analysed during the current study are available from the corresponding author on reasonable request.

## Abbreviations

MTPJ: Metatarsophalangeal joint
